# Exploring the neural link between childhood maltreatment and depression: a default mode network rs-fMRI study

**DOI:** 10.3389/fpsyt.2024.1450051

**Published:** 2024-09-13

**Authors:** Jian Lin, Jialing Huang, Yun Wu, Linqi Zhou, Changyuan Qiao, Jian Xie, Changchun Hu

**Affiliations:** ^1^ Department of Clinical Psychiatry, Affiliated Hangzhou First People’s Hospital, School of Medicine, Westlake University, Hangzhou, Zhejiang, China; ^2^ Department of Radiology, Affiliated Hangzhou First People’s Hospital, School of Medicine, Westlake University, Hangzhou, Zhejiang, China; ^3^ School of the Fourth Clinical Medicine, Zhejiang Chinese Medical University, Hangzhou, Zhejiang, China

**Keywords:** depression, childhood maltreatment, functional connectivity, independent component analysis (ICA), default mode network (DMN)

## Abstract

**Background:**

Childhood maltreatment (CM) is increasingly recognized as a significant risk factor for major depressive disorder (MDD), yet the neural mechanisms underlying the connection between CM and depression are not fully understood. This study aims to deepen our understanding of this relationship through neuroimaging, exploring how CM correlates with depression.

**Methods:**

The study included 56 MDD patients (33 with CM experiences and 23 without) and 23 healthy controls. Participants were assessed for depression severity, CM experiences, and underwent resting-state functional MRI scans. Independent Component Analysis was used to examine differences in functional connectivity (FC) within the Default Mode Network (DMN) among the groups.

**Results:**

MDD patients with CM experiences exhibited significantly stronger functional connectivity in the left Superior Frontal Gyrus (SFG) and right Anterior Cingulate Cortex (ACC) within the DMN compared to both MDD patients without CM experiences and healthy controls. FC in these regions positively correlated with Childhood Trauma Questionnaire scores. Receiver Operating Characteristic (ROC) curve analysis underscored the diagnostic value of FC in the SFG and ACC for identifying MDD related to CM. Additionally, MDD patients with CM experiences showed markedly reduced FC in the left medial Prefrontal Cortex (mPFC) relative to MDD patients without CM experiences, correlating negatively with Childhood Trauma Questionnaire scores.

**Conclusion:**

Our findings suggest that increased FC in the ACC and SFG within the DMN is associated with CM in MDD patients. This enhanced connectivity in these brain regions is key to understanding the predisposition to depression related to CM.

## 1 Introduction

Major depressive disorder (MDD) is a prevalent mental disorder characterized by emotional, cognitive, and physical symptoms, and it imposes a substantial economic burden on society (1, 2). Childhood maltreatment (CM) is recognized as a key risk factor for MDD, encompassing forms such as emotional abuse, emotional neglect, physical abuse, physical neglect, and sexual abuse (3). Approximately 46% of patients with depression report experiencing maltreatment during childhood (4). Additionally, CM has been consistently linked to poorer treatment outcomes in depression patients, significantly heightening the risk of relapse and suicide (5–7). It has been proposed that these associations result from the long-term adverse effects of CM on brain structure and function during critical periods of development (8, 9). These effects are particularly evident in brain networks associated with stress response, emotion processing, and self-referential processing, such as the Default Mode Network (DMN) (10). Therefore, elucidating the relationship between CM, the DMN, and depression is essential.

CM has been shown to impact the DMN, leading to altered connectivity patterns that may underlie the development of MDD (11). Specifically, the stress and trauma associated with CM are believed to disrupt the normal maturation of the DMN, resulting in abnormalities in self-referential processing and emotion regulation. These changes may further contribute to the core symptoms of MDD, such as emotional dysregulation, negative self-perception, and rumination (12).

Previous neuroimaging studies have found that CM in patients with depression is linked to structural and functional abnormalities in certain regions of the DMN, including the prefrontal cortex, cingulate gyrus, middle temporal gyrus, precuneus, and hippocampus (9, 13–18). Furthermore, a study on brain networks revealed that, compared to depression patients without CM and healthy controls, those with CM exhibited more pronounced reductions in functional connectivity between the DMN and the dorsal attention network (DAN), as well as between the ventral attention network (VAN) and the DMN. These findings suggest that by dysregulating the DMN, CM likely increases vulnerability to MDD.

In summary, there is a profound interrelation between CM, DMN, and depression. However, additional research is required to elucidate the biological mechanisms underlying the roles of CM and the DMN in the progression of depression. Most previous neuroimaging studies investigating brain function and structural changes in depression patients with a history of CM have relied on predefined regions of interest (ROIs) based on prior hypotheses. However, no studies to date have employed independent component analysis (ICA) to extract the DMN in depression patients and explore its relationship with CM. Unlike seed-based methods, ICA does not require predefined seed regions, allowing for the data-driven extraction of brain networks (19). Therefore, this study employs ICA to compare the differences in functional connectivity within the DMN network among patients with depression who have experienced CM, those who have not, and healthy participants. The aim is to explore the interrelation between CM, the DMN, and depression, thus shedding light on the intricate interplay of these factors.

## 2 Methods

### 2.1 Participants

This study was conducted with the approval of the Medical Ethics Committee of the First People’s Hospital of Hangzhou (IRB: 2020-K008-01). All participants were comprehensively informed about the study’s objectives and methods and provided informed consent via signed forms.

For patient inclusion, participants had to meet the following criteria (1): Diagnosis of depression according to DSM-IV criteria; (2) Ages ranging from 16 to 35 years; (3) Currently experiencing their first or a recurrent depressive episode, without having received antidepressant treatment within two weeks prior to enrollment; (4) No contraindications to undergoing MRI; (5) HAMD-17 score of 17 or above. Exclusion criteria included: (1) Current or past diagnosis of other Axis I mental disorders as per DSM-IV-TR; (2) Presence of severe or unstable physical illness; (3) pregnancy or current lactation. After enrollment, participants underwent face-to-face interviews to assess any history of CM. The Childhood Trauma Questionnaire (CTQ) was utilized to comprehensively document their experiences of maltreatment. Subsequently, patients were divided into two groups: those with depression related to CM (CM-MDD group) and those whose depression was not linked to such experiences (NCM-MDD group).

The study also included a Healthy Control group (HC), individuals recruited via online advertisements, and matched with the patient participants by age, gender, and educational attainment. Criteria for these participants were: (1) Age range from 16 to 35 years; (2) No history of mental disorders; (3) Absence of chronic physical illnesses; (4) No MRI contraindications; (5) CTQ score below the cutoff point on all five subscales.

In the imaging data processing phase, participants showing head motion exceeding 2mm or rotation angles beyond 2° were excluded. The finalized cohort thus consisted of 33 patients in the CM-MDD group, 23 in the NCM-MDD group, and 23 in the HC group.

### 2.2 Assessment

Participants underwent an extensive neuropsychological evaluation. The severity of depression was gauged using the 17-item Hamilton Rating Scale for Depression (HAMD-17) (20). Additionally, the Childhood Trauma Questionnaire (CTQ) (21), a 28-item retrospective self-report instrument, was utilized to assess experiences of CM. The CTQ encompasses five subscales: emotional abuse, emotional neglect, physical abuse, physical neglect, and sexual abuse. Subjects with CM were defined as having at least one of following: emotional abuse ≥ 13; physical abuse ≥ 10; sexual abuse ≥ 8; emotional neglect ≥ 15; physical neglect ≥ 10 (22).

### 2.3 MRI data acquisition and image preprocessing

Baseline fMRI data were collected utilizing a 3.0 Tesla Siemens Vireo scanner at Hangzhou First People’s Hospital, affiliate of the Xi Hu University School of Medicine. Participants were instructed to lie still, ensuring their eyes closed, and to avoid active thinking. This protocol standardized the resting state condition. Baseline fMRI data were collected using a 3.0 Tesla Siemens Vireo scanner at Hangzhou First People’s Hospital. Participants were instructed to remain still, with their eyes closed, and to avoid active thinking. This protocol standardized the resting-state condition.

The 3D-T1 structural images were acquired with the following parameters: Repetition time (TR), 1900 ms; Echo time (TE), 2.52 ms; inversion time (TI), 900 ms; Flip angle, 9°; Slice Thickness, 1 mm; gap, 0 mm; field of view (FOV), 256 × 256; and total scan duration, 4 minutes and 26 seconds. The 8-minute resting-state fMRI (rs-fMRI) scans, covered the entire brain over an 8-minute period, using a gradient-echo echoplanar imaging (GRE-EPI) sequence, with parameters set to: TR 2000 ms; TE 30 ms; Flip Angle 90°; Slice Thickness 3.2 mm; Total Slices 47; and FOV 220 × 220 mm.

Image preprocessing utilized the Data Processing Assistant for Resting-State fMRI 5.4 (DPARSF 5.4) in MATLAB R2020b (23). The initial 10 time points were excluded to ensure signal stability. This was followed by slice timing correction, motion (exceeded 2mm or where rotation surpassed 2°), spatial normalization to the MNI EPI template, and resampling to 3 × 3 × 3 mm voxels. Finally, spatial smoothing, using a 6mm Gaussian kernel, was then applied to reduce spatial noise.

### 2.4 Functional connectivity analyses

The preprocessed data underwent independent component analysis using the Group ICA of fMRI Toolbox (GIFT version 4.0b, mialab.mrn.org/software/gift). The minimum description length (MDL) criterion was adopted for data estimation, identifying 27 as the optimal number of independent components (24). Principal component analysis and the infomax algorithm facilitated data dimensionality reduction, executed 100 times for assured stability of group ICA components. Each participant’s data were decomposed, reconstructed into 27 distinct spatial components, and subsequently converted to Z-scores. Informed by prior studies offering DMN templates (25), a visual assessment and selection process of DMN results was performed.

### 2.5 Statistical analysis

Demographic and clinical characteristics of the participants were analyzed using the Statistical Package for the Social Sciences version 26.0 (SPSS 26.0; IBM, Armonk, NY, USA). Neuroimaging data analyses were conducted using the Data Processing Assistant for Resting-State fMRI (DPARSFA) software. The statistical analyses encompassed five primary domains:

1) Behavioral Analysis: Group differences were explored using T-tests, chi-square (χ2) tests, or one-way ANOVAs, supplemented by *post-hoc* Bonferroni corrections for multiple comparisons.

2) Default Network Extraction: Default network images for each group were identified and extracted through a one-sample t-test applied to selected independent components (ICs). Gaussian Random Field (GRF) correction for multiple comparisons was employed, maintaining a cluster significance threshold of *p* < 0.05.

3) Functional Connectivity: Differences in network connectivity among groups were assessed using a one-way ANOVA on a network mask generated from the one-sample t-test. Significant regions, post one-way ANOVA, were subjected to *post-hoc* multiple comparisons (Voxel *p* < 0.001, Cluster *p* < 0.05, GRF-corrected).

4) Correlation Analysis: The association between abnormal DMN brain regions zFC and CTQ score in MDD patients was elucidated using partial correlation analysis.

5) Receiver Operating Characteristic (ROC) Curve: ROC curve analysis was utilized to evaluate the predictive value of zFC in differentiating brain regions between CM-MDD and NCM-MDD. The area under the curve (AUC) was calculated to assess the classification potential between these groups.

## 3 Results

### 3.1 Demographics and clinical features

Statistical analysis revealed no significant differences in gender, age, and educational attainment among the MDD-CM group, MDD-NCM group, and the HC group (*p* > 0.05). However, notable discrepancies were observed in the scores of the HAMD-17 and both the total and subscale scores of CTQ across these groups (**Table 1**).

**Table 1:** Comparison of demographic and clinical data among subjects.

Variables	Group			*F/x^2^*	*p-*value	*Post-hoc*
	CM-MDD(*n*=33)	NCM-MDD (*n*=23)	HC (*n*=23)			
Age(years)	20.81 ± 6.03	19.30 ± 4.79	20.87 ± 3.48	0.755	0.473^b^	–
Gender(M/F)	6/27	10/13	10/13	5.569	0.062^a^	–
Education(years)	12.79 ± 2.45	12.61 ± 2.29	13.90 ± 2.83	1.744	0.182^b^	–
HAMD-17	20.70 ± 3.92	21.22 ± 4.17	0.70 ± 1.30	278.444	<0.001^b^	CM-MDD&NCM-MDD>HC
CTQ score	61.27 ± 12.20	36.39 ± 6.07	31.57 ± 6.89	83.814	<0.001^b^	CM-MDD>NCM-MDD&HC
Emotional abuse	14.64 ± 4.26	8.78 ± 2.58	6.35 ± 1.82	48.829	<0.001^b^	CM-MDD>NCM-MDD&HC
Physical abuse	8.97 ± 3.64	6.09 ± 1.28	5.83 ± 1.59	12.903	<0.001^b^	CM-MDD>NCM-MDD&HC
Sexual abuse	7.09 ± 3.31	5.30 ± 0.64	5.09 ± 0.42	7.274	0.01^b^	CM-MDD>NCM-MDD&HC
Emotional neglect	18.33 ± 3.52	9.78 ± 2.97	8.09 ± 3.70	73.793	<0.001^b^	CM-MDD>NCM-MDD&HC
Physical neglect	12.24 ± 3.26	6.43 ± 1.27	6.22 ± 1.70	58.184	<0.001^b^	CM-MDD>NCM-MDD&HC

CM, childhood maltreatment; NCM, Non-childhood-maltreatment; MDD, major depressive disorder; HC, healthy controls; HAMD-17, 17-item Hamilton Depression Rating Scale. ^a^ Chi-square test; ^b^ One-way ANOVA.

### 3.2 DMN selection

**Figure 1:**
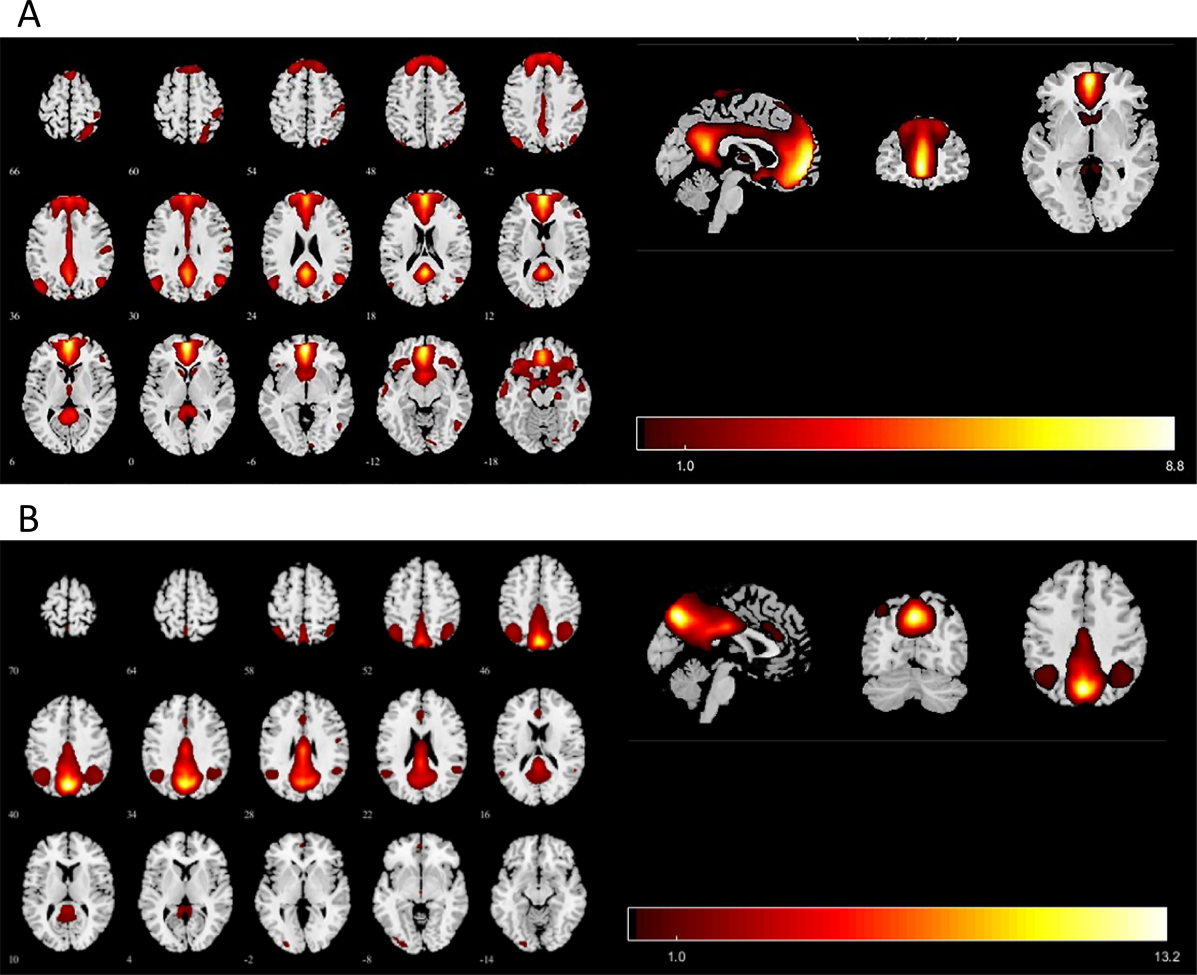
Images showing the DMN extracted by independent component analyses in all subjects. **(A)** The IC identified as aDMN. **(B)** The IC identified as pDMN. IC, independent component; aDMN, anterior default-mode network; pDMN, posterior default mode networks. *p* < 0.001 at the voxel level, *p* < 0.05 at the cluster level, GRF multiple comparison correction.

The 27 ICs decomposed by ICA were visually inspected against the default network template from previous studies, and two subnetworks of the default network were identified through manual selection, including the anterior default network (aDMN) and the posterior default network (pDMN) (**Figures 1A**, **B**).

### 3.3 Statistic comparison of intra-FC values differences between groups in DMN

**Figure 2:**
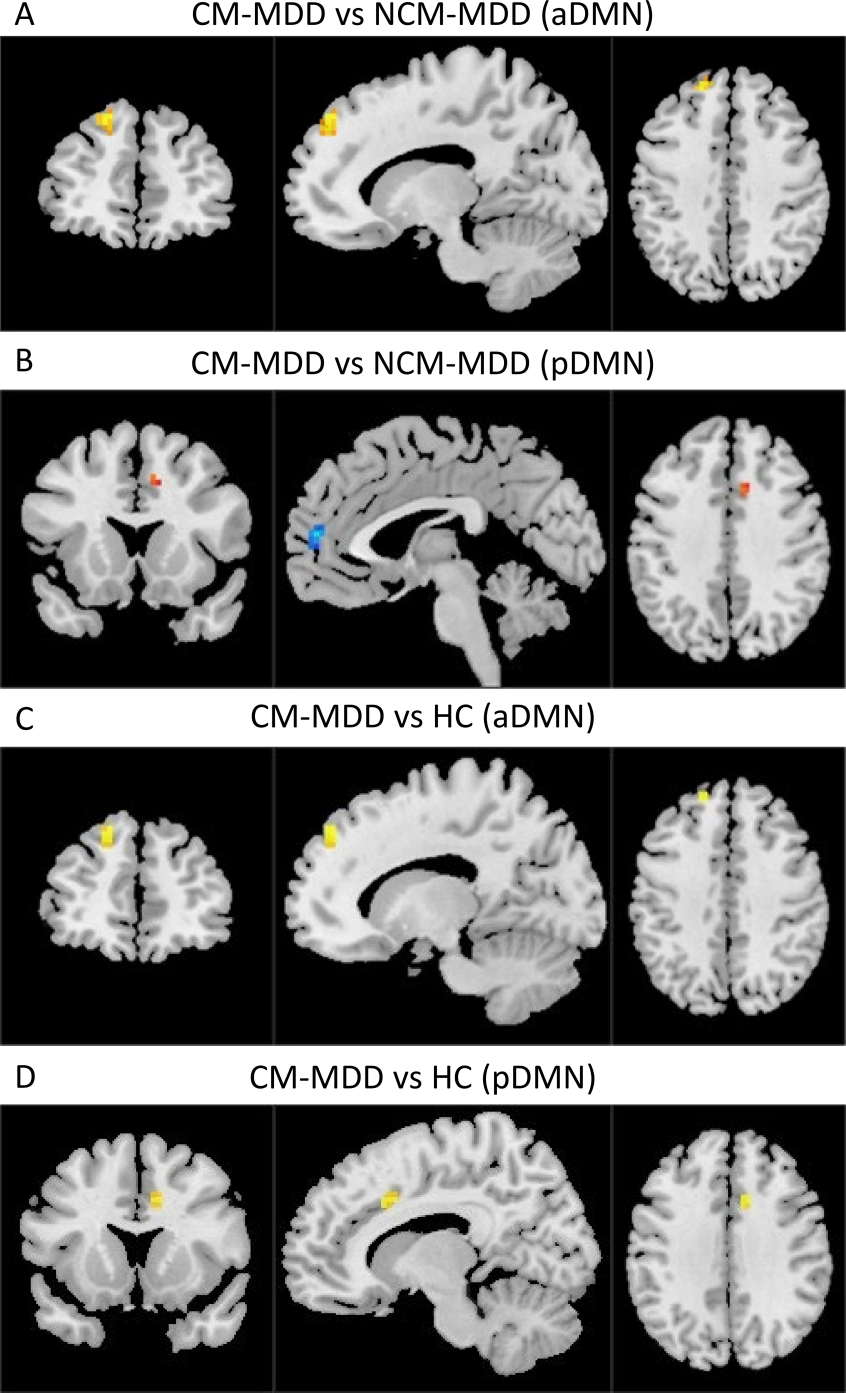
The brain regions with FC differences within RSNs are located in the aDMN and pDMN (GRF multiple comparison correction, voxel-level p-values < 0.001, cluster-level p-values < 0.05). Warm colors represent areas with increased FC compared to the two groups, while cool colors represent areas with weakened FC. **(A)** Comparison between CM-MDD and NCM-MDD groups showing increased FC in the left SFG within the aDMN. **(B)** Comparison between CM-MDD and NCM-MDD groups showing decreased FC in the left mPFC within the pDMN and increased FC in the right ACC. **(C)** Comparison between CM-MDD and HC groups showing enhanced FC in the left SFG of the aDMN. **(D)** Comparison between CM-MDD and HC groups showing increased FC in the right ACC of the pDMN.

In the group-level functional connectivity analysis, compared to the NCM-MDD group, the CM-MDD group demonstrated increased connectivity in the left superior frontal gyrus (SFG) within the aDMN (Peak MNI coordinates: X = -15; Y= 48; Z = 42; cluster size =25, *t* = 5.793, *Cohen’s f^2^* = 0.62) (**Figure 2A**). Meanwhile, connectivity in the left medial prefrontal cortex (mPFC) within the pDMN (Peak MNI coordinates: X = -3; Y= 54; Z = 12; cluster size =16, *t* = -5.385, *Cohen’s f^2^* = 0.53) was decreased, and connectivity in the right anterior cingulate cortex (ACC) was increased (Peak MNI coordinates: X = 9; Y= 15; Z = 39; cluster size =10, *t* = 4.012, *Cohen’s f^2^* = 0.30) (**Figure 2B**). Compared to the healthy control (HC) group, the CM-MDD group exhibited enhanced functional connectivity in the left SFG of the aDMN (Peak MNI coordinates: X = -15; Y= 48; Z = 42; cluster size =14, *t* = 4.598, *Cohen’s f^2^* = 0.39) (**Figure 2C**) and the right ACC of the pDMN (Peak MNI coordinates: X = 9; Y= 15; Z = 36; cluster size =18, *t* = 5.051, *Cohen’s f^2^* = 0.47) (**Figure 2D**). No significant differences were observed between the NCM-MDD group and the HC group after multiple comparison correction. These results were corrected using GRF for multiple comparisons (voxel-wise *p* < 0.001, cluster-level *p* < 0.05) (**Table 2**).

**Table 2:** Brain areas with significant differences in the intra-FC values among three group.

Contrast	RSNs	Cluster	MNI			*t* value	Voxel size	Effect size (*Cohen’s f^2^*)
			X	Y	Z			
CM-MDD vs NCM-MDD								
	aDMN	L_SFG	-15	48	42	5.793	25	0.62
	pDMN	L_MPFC	-3	54	12	-5.358	16	0.53
	pDMN	R_ACC	9	15	39	4.012	10	0.30
CM-MDD vs HC								
	aDMN	L_SFG	-15	48	42	4.598	14	0.39
	pDMN	R_ACC	9	15	36	5.051	18	0.47

*p* < 0.001 at the voxel level, *p* < 0.05 at the cluster level, GRF multiple comparison correction.

### 3.4 Intra-FC changes in MDD patients and their relation to CTQ scores

**Figure 3:**
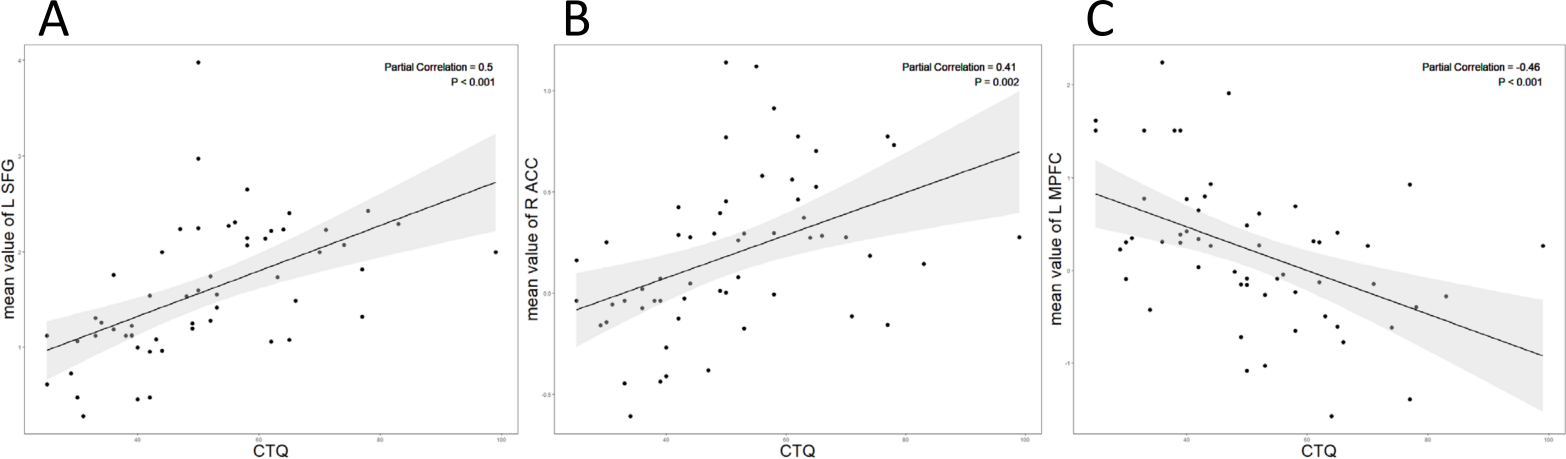
**(A)** The CTQ score showed a positive correlation with the intranetwork FC values of the left SFG (*r* = 0.50, *p*<0.001); **(B)** The CTQ score showed a positive correlation with the intranetwork FC values of the right ACC (*r* = 0.41, *p*= 0.002). **(C)** The CTQ score showed a negative correlation with the intranetwork FC values of the left mPFC (*r* = -0.46, *p*<0.001).

Partial correlation analysis revealed a positive association between zFC in the left SFG (*r* = 0.50, *p* < 0.001, **Figure 3A**) and right ACC (*r* = 0.41, *p* = 0.002, **Figure 3B**) within DMN network and scores on the CTQ. Conversely, zFC in the left mPFC showed a negative correlation with CTQ scores (*r* = -0.46, *p* < 0.001, **Figure 3C**). Age and gender were controlled for as covariates in the analysis.

### 3.5 ROC curve analysis

**Figure 4:**
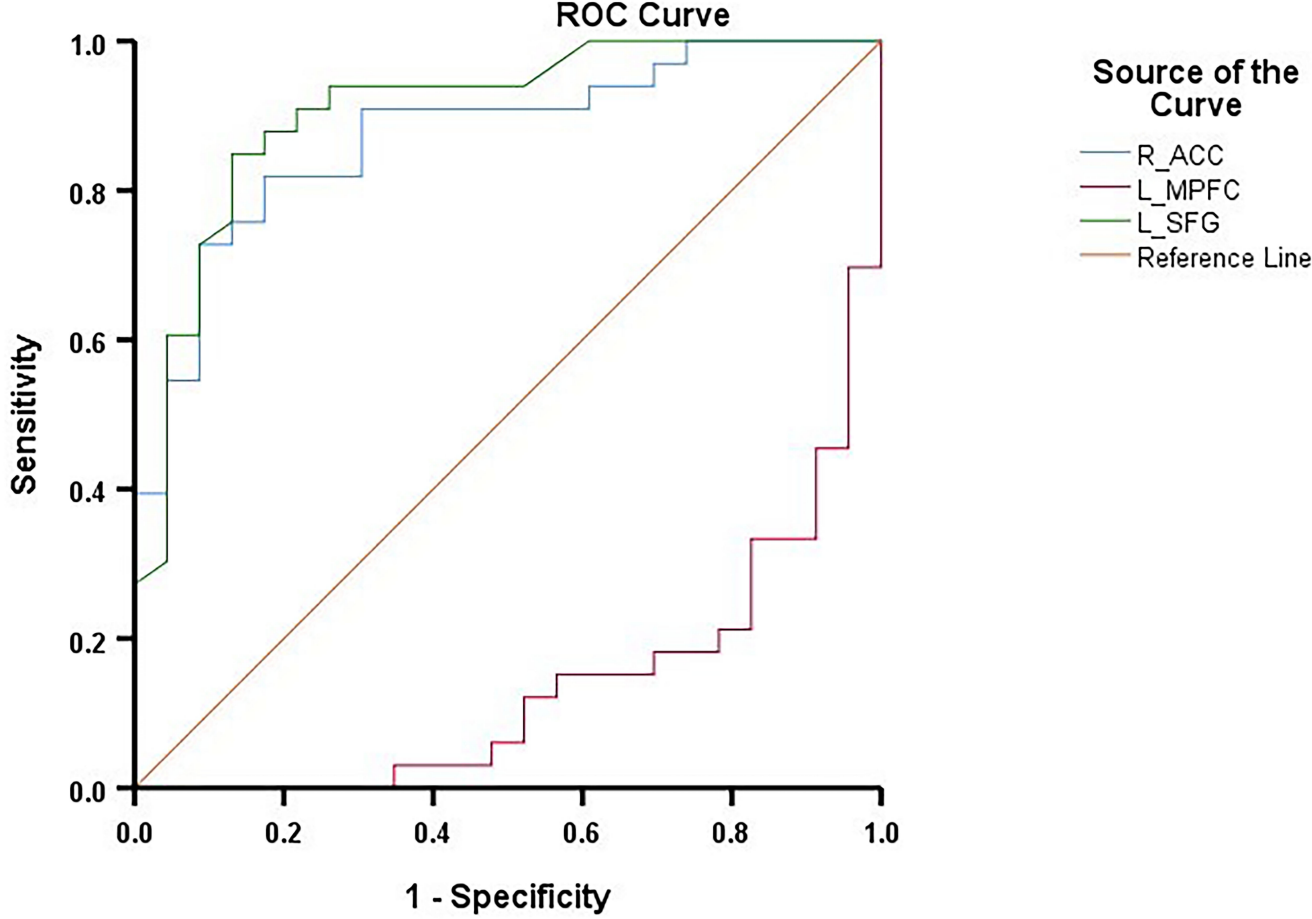
ROC curves for differentiating the CM-MDD group and the NCM-MDD group. ROC, receiver operating characteristic; AUC, area under the curve.

In the ROC curve analysis (**Figure 4**), The FC values showed significant discriminative power between the CM-MDD and NCM-MDD groups. Specifically, the AUC for FC between the SFG and DMN was 0.906 (95% CI: 0.825-0.988, *p* < 0.001), while for FC between the right ACC and DMN, the AUC was 0.847 (95% CI: 0.782-0.965, *p* < 0.001). For FC between the left MPFC and DMN, the AUC was notably lower at 0.136 (95% CI: 0.041-0.230, *p* < 0.001).

## 4 Discussions

In this study, we employed ICA, a data-driven approach, to investigate the association between the intra-FC of the DMN and CM in patients with MDD. Notably, the CM-MDD group, as compared to both the NCM-MDD and HC groups, demonstrated increased functional connectivity between the left SFG and the right ACC within the DMN. This increase correlated positively with CTQ scores. ROC curve analysis indicated that the intra-network functional connectivity between the left SFG and the right ACC effectively distinguished between the CM-MDD and NCM-MDD groups. Additionally, the CM-MDD group, in comparison to the NCM-MDD group, showed decreased functional connectivity in the left mPFC within the DMN, which negatively correlated with CTQ scores. These findings augment our understanding of the neuroimaging mechanisms that link CM and MDD, aspects of which we will further discuss and analyze below.

In this study, enhanced FC was observed in both the left SFG and the right ACC within the DMN in MDD patients with CM. Building upon a prior study that linked CM and increased FC in the SFG to anxiety and attention impulsivity (26), our results extend this correlation to a clinical MDD population. This suggests a neurobiological pathway potentially contributing to depression onset. The SFG’s involvement in cognitive behavior, decision making, and emotion regulation (27–29) underscores its possible role as a mediator in the CM-MDD relationship. Our findings of altered FC in the ACC, a region implicated in cognitive behavior, sensation processing, and emotion regulation (30–32), further corroborate this link. Studies indicating reduced ACC volume in CM-experiencing healthy individuals (8, 13, 33, 34) hint at a strong ACC-CM connection. Reduced ACC volume in MDD patients with CM (35) suggests an intertwined relationship between CM and MDD through the ACC. Aligning with our findings, a resting-state fMRI study showed increased bilateral FC between the lateral ACC and the MFG in patients with moderate to severe CM (36). Intriguingly, task-state fMRI research (17) revealed differential ACC activation in self-processing tasks among CM individuals, emphasizing the ACC’s vital role in MDD’s emotional and cognitive dysfunctions. The observed connectivity enhancement in CM contexts might indicate compensatory neural responses or maladaptive changes related to MDD pathophysiology.

The mPFC, a crucial component of the DMN, is linked with self-reflection, self-awareness, and autobiographical memory (37, 38). Our study discovered that, in comparison to the NCM-MDD group, the CM-MDD group exhibited a marked reduction in FC in the left mPFC, which negatively correlated with CTQ scores. These findings suggest a strong link between the mPFC and CM. However, this effect was not observed when comparing MDD patients with healthy controls. It’s important to note that this result is not consistent across all studies. for instance, a previous study (39) reported a decrease in mPFC FC in MDD patients with CM as compared to those without CM and healthy controls, significantly related to childhood neglect measures. Furthermore, other research (16) noted a notable effect of the interplay between MDD diagnosis and CM on rs-FC in certain DMN areas, including the bilateral mPFC. Subsequent analysis indicated that MDD patients with moderate to severe CM, as opposed to those with no or low CM, showed reduced bilateral mPFC rs-FC, suggesting that DMN FC changes are associated with CM exposure in MDD patients. This difference in medication status could affect functional connectivity in MDD patients and may partially explain the differences observed when compared to healthy controls. Furthermore, differences in MRI scanning parameters and analytical methods may also contribute to these subtle discrepancies.

Additionally, according to previous recommendations (40), an AUC value greater than 0.7 indicates an acceptable discriminatory ability of a biomarker/test for diagnosing disease. ROC curve analysis in this study demonstrates that FC within the DMN, particularly in the left SFG and right ACC, can effectively distinguish between the CM-MDD and NCM-MDD groups, showing significant diagnostic and classification capabilities. This finding suggests that functional connectivity within the DMN, identified through ICA analysis, can serve as a biomarker for differentiating depression cases with and without CM, implying a potential link between the DMN, CM, and depression. However, the application of more precise techniques, such as machine learning methods, requires further exploration.

SFG and ACC are anatomically interconnected and both play crucial roles in emotional regulation and cognitive behaviors (41). Notably, prior research has highlighted the mediating role of maladaptive cognitive processes, such as rumination, and difficulties in emotional regulation in the nexus between CM and depression (42–44). Consequently, we hypothesize that abnormal functional connectivity within the DMN, particularly involving the SFG and ACC, may contribute to maladaptive cognitive processes and impaired emotional regulation strategies, thereby increasing the risk of depression development. Previous neuroimaging studies investigating the relationship between CM and depression have predominantly relied on seed-based analyses. In contrast, our study utilized ICA to further elucidate the role of the DMN in CM-induced depression. This approach offers a novel perspective on the involvement of the DMN in the development of depression following CM. Future longitudinal studies are likely needed to further validate this relationship.

While this study provides valuable insights, several limitations warrant consideration in interpreting the findings: 1) Participant History: Including participants with recurrent depressive disorders and prior antidepressant treatment may have influenced DMN functional connectivity. These factors could introduce DMN variability unrelated to CM. 2) Sample Size: The relatively small sample size could lead to false-negative or false-positive results. Larger studies are necessary to confirm these findings and ensure their generalizability. The difference in sample sizes between the CM-MDD and NCM-MDD groups could potentially impact the study’s results. we use of GRF correction and effect size reporting ensures that our results maintain statistical power despite the difference in sample sizes, making the findings more reliable. 3) Retrospective Reporting of CM: The reliance on retrospective interviews to assess CM might result in recall biases. The accuracy of reported data could be affected by various factors, including the current mental state. 4) Cross-sectional Design: The study’s cross-sectional nature limits the ability to establish causal relationships between DMN abnormalities and MDD development. Longitudinal studies are needed to clarify these associations’ directionality. 5) Control Group: The lack of a healthy control group with a history of CM significantly limits the study. Such a group would have provided deeper insights into CM’s impact on DMN connectivity independent of MDD. 6) CM Subtypes: We did not explore potential differences in DMN functional connectivity among MDD patients with different types of CM. Our current sample size was insufficient to perform meaningful subgroup comparisons. Future studies with larger sample sizes are needed to investigate these distinctions and better understand the specific impacts of various CM subtypes on DMN connectivity.

## 5 Conclusion

In summary, our findings suggest that childhood abuse is linked to functional connectivity abnormalities within the DMN of patients with severe depression. Specifically, the FC in the left SFG and right ACC effectively differentiates between CM-MDD and NCM-MDD. These insights could facilitate a deeper understanding of the DMN’s specific role in MDD pathophysiology and the neurophysiological connection between CM and MDD. This lays a foundation for further investigation into early intervention and targeted treatment strategies for those affected.

## Data Availability

The raw data supporting the conclusions of this article will be made available by the authors, without undue reservation.
